# SHANK3 and beta-synuclein are novel blood-based biomarkers for the Phelan-McDermid Syndrome: a pilot study

**DOI:** 10.1038/s41398-026-03932-8

**Published:** 2026-03-24

**Authors:** Jessica Pagano, Andrea Perez Arevalo, Anastasia Nosanova, Helen Friedericke Bauer, Eva Loth, Stephanie Andres, Felicitas Becker, Markus Otto, Alessia Stefanoni, Chiara Verpelli, Patrick Oeckl, Michael Schön, Tobias Boeckers

**Affiliations:** 1 Institute for Anatomy and Cell Biology, Ulm University, 89081 Ulm, Germany; 2 Clinic of Neurology, Ulm University, 89081 Ulm, Germany; 3 Institute of Psychiatry, Psychology and Neuroscience (IoPPN), King’s College London, London, UK; 4 Medicover München Ost MVZ, 81667 München, Germany; 5 Clinic of Neurology, Halle University, 06120 Halle, Germany; 6 CNR, Institute of Neuroscience, Milan, Italy; 7 DZNE, Ulm site, 89081 Ulm, Germany

**Keywords:** Diagnostic markers, Molecular neuroscience

## Abstract

Phelan-McDermid syndrome (PMS) is a relatively frequent cause of syndromic intellectual disability (ID) and autism spectrum disorder (ASD). It is typically caused by genetic alterations in the 22q13 chromosomal region, most often involving heterozygous deletions or mutations in the *SHANK3* gene. More than half of affected individuals exhibit functional impairments in speech, cognition, motor skills, and behavior. Despite multiple ongoing therapeutic programs, objective and scalable liquid biomarkers to support patient stratification and to monitor disease course or treatment response are still lacking. Here, in a pilot study involving 23 individuals with PMS, we identified two biomarkers that are significantly altered compared to a control group and are associated with symptom severity. First, SHANK3 protein was detectable in peripheral blood mononuclear cells (PBMCs) and was markedly reduced in PMS (mean −77% vs. controls), consistent with SHANK3 haploinsufficiency; lower PBMC SHANK3 levels were associated with the presence of developmental regression, supporting its potential utility as a target-engagement/monitoring biomarker rather than a diagnostic screen. Additionally, plasma levels of beta-synuclein, a neuron-specific synaptic protein, were elevated in PMS and positively correlated with the severity of speech impairment. Both biomarkers were successfully back-translated in a *Shank3* transgenic mouse model, where beta-synuclein levels were normalized through modulation of the mGlu5 receptor. Together, these results provide initial evidence for SHANK3 in PBMCs and plasma beta-synuclein as complementary liquid biomarkers to aid prognosis and enable objective monitoring of therapeutic response in PMS, warranting validation in larger and pediatric longitudinal cohorts.

## Introduction

Phelan-McDermid Syndrome (PMS) is a complex neurodevelopmental disorder classified as a syndromic form of autism spectrum disorder (ASD) [1]. The core clinical features of PMS include neonatal hypotonia, dysmorphic features, global developmental delay, absent or severely impaired speech, intellectual disability, seizures, and ASD associated behavioural phenotypes [1–3]. PMS results from a deletion on the distal long arm of chromosome 22, specifically the 22q13.3 region, with deletion sizes ranging from less than 100 kb to more than 9 Mb [4]. This deletion can also be the result of a ring chromosome 22. In nearly all these cases, the *SHANK3* gene is affected. Moreover, another subgroup of patients with PMS carries a pathologic *SHANK3* variant or has breakpoint mutation within the gene [4, 5]. Consequently, SHANK3 haploinsufficiency is widely considered the principal driver of the neurological phenotype [1]. Rare cases have been described in which the deletion does not include SHANK3 [6–10], underscoring both the genetic heterogeneity of 22q13 alterations and the clinical variability across the PMS spectrum. Importantly, PMS is estimated to occur at approximately 1 in 15,000–20,000 individuals, placing it among the more prevalent single-gene/defined-etiology contributors to syndromic neurodevelopmental disability.

*SHANK3* encodes a key scaffolding protein primarily localized at the postsynaptic density (PSD) of excitatory synapses. There, it plays a critical role in modulating synaptic maturation and function, also by linking surface receptors and other PSD proteins to the actin cytoskeleton [11–13]. Notably, SHANK3 expression has also been detected in glial cells such as oligodendrocytes [14] and significant white matter changes have been described in PMS individuals as well as in Sh*ank3* mouse models [15]. Alterations in *SHANK3* have been strongly linked to ASD in numerous studies [16–18]. It is estimated that *SHANK3* mutations or deletions account for approximately 1% of all ASD cases [16].

While the molecular diagnosis of PMS is straightforward once genetic testing is performed. However, due to the variability in symptom presentation and the often non-specific clinical phenotype, PMS remains underdiagnosed [5, 10, 15, 19]. a major unmet need lies elsewhere: the field lacks objective, scalable liquid biomarkers that can support patient stratification, inform prognosis, and—critically—provide monitoring and pharmacodynamic endpoints for therapeutic trials. This need is increasingly pressing given the growing number of academic and industry-led programs in PMS, including approaches aimed at synaptic function, neurotrophic pathways, and emerging gene-based strategies. Current trial endpoints often rely on behavioral assessments and other measures that can be variable, age-dependent, and sensitive to rater and contextual effects. Biomarkers that can be repeatedly sampled with minimal burden would therefore be valuable, not as diagnostic screening tools, but as measures of disease biology and treatment response over time.

In this study, we examined whether peripheral SHANK3 expression could be detected and leveraged as a blood-based marker, motivated by the central role of SHANK3 in PMS and the practical advantages of peripheral sampling. We assessed SHANK3 mRNA and protein in peripheral blood mononuclear cells (PBMCs) from a well-characterized Shank3 knockout mouse model [20] (Shank3Δ11 − /−) and from individuals with PMS and matched controls. In parallel, we investigated beta-synuclein as an additional candidate blood marker. This selection was hypothesis-driven: beta-synuclein is a neuron-enriched synaptic protein measurable in blood using sensitive targeted proteomic methodologies and has been implicated as a marker of synaptic dysfunction in other neurological contexts, making it a plausible readout to explore in a SHANK3-related synaptopathy [21–25].

Finally, to connect molecular measures with clinically meaningful features and to evaluate therapeutic responsiveness in a translational framework, we examined associations between these biomarkers and phenotypic characteristics in the human cohort and tested treatment-related modulation in the Shank3 mouse model using mGlu5 receptor modulation [26]. mGlu5 signaling is mechanistically linked to synaptic scaffolding [12, 13, 26, 27] and has shown beneficial effects in Shank3-related models, providing a rationale to probe whether a candidate liquid marker might also serve as a pharmacodynamic readout. Collectively, our approach aims to establish feasibility for two complementary blood-based measures—PBMC SHANK3 as a potential target-engagement/monitoring marker and plasma beta-synuclein as a candidate marker associated with clinical features and treatment response—thereby supporting future longitudinal and pediatric validation studies.

## Materials/subjects and methods

### Ethical statement

Informed consent was obtained from all human donors or their legal guardians prior to sampling. All experiments with human participants were conducted in compliance with national law and found to be approved by the local ethics committee in Ulm, Germany (no. 124/23 for healthy control individuals, no. 321/16 for individuals with PMS). All experiments with mice have been conducted according to national and EU law and approved by Regierungspräsidium Tübingen (Nr. o. 103-17).

### Mice

The *Shank3*Δ11-/- (referred as *Shank3* KO) mice were previously described (Schmeisser et al., 2012). *Shank3* fullKO mice were purchased from Jackson Laboratory. All mice were housed under standard laboratory conditions, with access to food and water ad libitum, and a 12 h dark/light cycle. WT, *Shank3*Δ11 + /– and *Shank3*Δ11–/– mice used in this study were obtained from heterozygous breeding on a C57BL/6 background. Animal from both sexes were included in all experiments. All animal experiments were conducted in compliance with the guidelines for the welfare of experimental animals issued by the Federal Government of Germany, and approved by the Ethics Committee of Regierungspräsidium Tübingen, (Nr. o. 103-17).

### Mice blood collection, plasma and PBMC isolation

Adult mice were anesthetized by inhalation of CO2. Blood was collected via cardiac puncture and the mice were sacrifice by decapitation. The blood was immediately placed in an EDTA-KE tube (Sarstedt 061664001) and processed.

For PBMCs isolation, the collected blood was diluted 1:1 with PBS -/- + 2% FBS solution at room temperature. The right amount of Lymphoprep Density Gradient Solution (Cat. 07905) was add to a fresh tube and the diluted blood was gently layered on top of the density gradient medium. Samples were centrifuged at 800 g for 30 min at room temperature. Carefully, cells were harvested and washed in PBS -/- + 2% FBS solution. After a centrifugation at 3000 rpm for 10 min at room temperature, the supernatant was discarded and the pellet was processed for further uses.

For Plasma isolation, the whole blood was centrifuged at 2000 g for 15 min at 4 °C.

### Human blood collection, plasma and PBMC isolation

Blood collection for individuals with PMS was performed at one afternoon during a family gathering. Families were contacted prior to the meeting and also informed about the study during two medical-scientific presentations prior to blood collection. Preference was given to recruiting adolescents and adults, and younger children were recruited if it was known that they tolerated blood drawing well. Two physicians performed the blood collections. Acute and chronic infections or inflammatory conditions were considered as exclusion criteria. Controls were recruited among Ulm university. The same exclusion criteria were applied, and participants did not report any neurological or mental illnesses.

Sample size was determined by availability rather than an a priori power calculation because PMS is a rare disorder, and we enrolled all eligible participants available during the family gathering. We then performed a sensitivity (MDE) analysis showing that the group sizes provide ~80% power (two-sided α = 0.05) to detect moderate to large standardized group differences (Cohen’s d ~ 0.9). In covariate-adjusted OLS models, biomarkers were z-scored and standardized effects are reported directly as the coefficient in SD units (with confidence intervals).

Human whole blood was collected using the BD Vacutainer CPT cell preparation tube with sodium citrate (Ref. 362761). After collection, tubes are gently inverted and cenrifuged at 1800g for 30 min at room temperature. After centrifugation plasma was collected and stored at −80 °C. Mononuclear cells are collected, washed in PBS -/- + 2% FBS solution and centrifuged again at 3000 rpm for 10 min at room temperature. After the centrifugation, supernatant is discarded and the pellet is processed for further uses.

### Biochemistry and quantitative immunoblot analysis

PBMCs were resuspended in Modified RIPA Buffer (10 mM TrisHcl pH 7,4, 1% Triton X-100, 0,1% SDS, 1% Sodium Deoxycholate, 5 mM EDTA) with phosphatase (PhosphoSTOP™, Roche 04906837) and protease Inhibitor (Complete™ Mini EDTA-free Protease Inhibitor Cocktail, Merck 11873580001). Cells were homogenizated using a 26 G needle and ultrasound (10 pulses). Samples were left in ice for 15 min and subsequently cell debris were separated from the protein lysate by a centrifugation step at 13.000 rpm for 10 min at 4 °C. Finally, supernatant was collected and stored at −80 °C or processed for further uses.

Protein concentration of cell lysates was calculated using the Bradford Assay and the signal intensity was measured using the Cytation3 ELISA reader. All samples were read in duplicates and the protein concentration was calculated in Microsoft Excel based on a standard curve previously calculated.

Equal amounts of each sample (40 µg) were separated using 4–20% gradients Mini-PROTEAN TGX Free-Stain precast gels.

After electrophoresis, the gel was activated for 45 sec using the ChemiDoc MP from BioRad. Protein was blotted on nitrocellulose membranes using the Trans-Blot Turbo Transfer System (BioRad). After the transfer, the total protein was imaged using the ChemiDoc MP machine and the membrane was blocked with 5% BSA in Tris-buffered saline containing 0.2% Tween-20 (TBST 0.2%) for 4 h at RT on a shacker. Membranes were incubated overnight at 4 °C with primary antibody in blocking solution. The folowing primary antibodies were used: SHANK3, cell signaling technologies, 64555; TREM2, ReD, MAB1729; TREM2, Cell Signaling Technologies, 91068; DAP12, Abcam, 283679; DAP12, Santa Cruz, sc133174; CD3, Abcam, AB11089.

Subsequently, membranes were washed 3 times with TBST 0.2% and incubated with horseradish peroxidase (HRP)-conjugated secondary antibodies (Dako) diluted in blocking solution for 1 h at RT. Afterward, membranes were washed 3 times with TBST 0.2% and the signal was measured using the Clarity Western ECL Substrate (BioRad 1705061) in the ChemiDoc MP machine.

All signals were quantified using ImageLab software (Biorad) and normalized against the value of the respective signal for total protein.

### Quantitative real-time PCR

Isolation of total RNA was performed using the RNeasy Mini kit (Qiagen) as described by the manufacturer and eluted in a total of 30 μl RNAse-free water.

Quantitative real-time reverse transcription PCR (qRT–PCR) was carried out in a one-step, single-tube format using the QuantinNova SYBR Green RT-PCR kit (Qiagen).

Thermal cycling and fluorescence detection were performed using the Rotor-Gene-Q real-time PCR machine model 2-Plex HRM (Qiagen). The qRT-PCR was assayed in 0.1 ml strip tubes in a total volume of 20 μl reaction mixture containing 1 μl of RNA, 2 μl of oligonucleotides, 10 μl of QuantiNova SYBR Green Master Mix, 6.8 μl of RNase-free water and 0.2 μl of Reverse Transcription Mix. The used validated primers were purchased from Qiagen (Mm_Gapdh_3_SG QT01658692; Hs_SHANK3_2_SG QT01851801; Hs_GAPDH_1_SG QT00079247) or Eurofins (Shank3 fw 5’-CCAAGTTCATCGCTGTGAAGG - 3’; rev 5’- TGTCATACTGTCGCATCTGCA - 3’).

Resulting data was analyzed utilizing the GAPDH gene to normalize transcript levels.

Cycle threshold (ct) values were calculated by the Rotor- Gene-Q Software (version 2.0.2, Qiagen). All reactions were run in technical duplicates, and mean ct values for each reaction were taken into account for data analysis.

### Blood smear staining

A drop of blood was placed on the slide (Fisher Scientific 16069901) and spreaded by using another slide placed at a 45° angle. Air-dried smears were fixed for 1 min in methanol. Smears were air-dried and washed three times with PBS (Gibco). Subsequently, slides were incubated with primary antibody (SHANK3 cell signaling 64555, 1:100) for 1 h at room temperature. After that two washes with PBS were performed and blood samples were incubated with AlexaFluor-conjugated secondary antibodies (Thermo Fisher Scientific, 1:250) for 1 h at room temperature. Finally, two washes with PBS were performed and slides were mounting using the ProLong Gold antifade reagent with DAPI (Thermo Fisher Scientific P36935).

### Plasma beta-synuclein analysis

Beta-synuclein concentration in human plasma samples was quantified as previously described [21, 22]. In brief, beta-synuclein was immunoprecipitated from 500 µL plasma using a monoclonal anti-beta-synuclein antibody (EP1537Y, Abcam) and quantified using multiple reaction monitoring mass spectrometry (IP-MS). An external calibration curve was generated using recombinant full-length beta-synuclein (rPeptide) at concentrations ranging from 2–100 pg/mL and 15N-labeled beta-synuclein was included in all samples as internal standard. Samples were analyzed in two runs and quality control samples (low and high) were included to monitor the performance of measurements (intra- and interassay CVs were 2.9–10.9 and 3.8–14.4%).

Since the required volume of 500 µl plasma for the validated IP-MS method is not applicable to murine studies and preliminary data indicated that beta-synuclein plasma levels in mice are substantially higher than in humans, we here exploratorily used an beta-synuclein immunoassay for the microfluidic Ella platform (Simple Plex Human beta-synuclein Cartridge from Biotechne) to quantify beta-synuclein in murine plasma samples. We tested the suitability of this assay for measurement in murine plasma samples in a pilot experiment using a pooled mouse plasma sample. The intraassay CVs for murine plasma QC samples was 5.3–8.3%.

### Pharmacological treatment

VU0409551 (Tocris 5693) was resuspended in a betaciclodextrin 20% (Sigma 332607) solution. *Shank3* WT and KO mice received an intraperitoneal injection of VU (5 mg/Kg) or vehicle for 10 days. Blood was collected 30 min after the last injection.

### Genetic reports and phenotype data

The survey included questions on the following binary or ordinal scaled phenotypes: speech (none/single words/sentences), gait disorder, muscle weakness (none/mild/severe), epilepsy (none/one seizure/multiple seizures), use of epilepsy medication, regression, cognitive impairment (none or mild/moderate/severe), behavioral problems (autism/hyperactivity or ADHD/catatonia/sleep disorder/anxiety disorder/depression or mania or bipolar disorder), therapy with intranasal insulin (none/previous use/current use). Parents or legal guardians answered the questions. Respondents could contact the study director on site in person at any time if they had any questions while completing the questionnaire. The survey was generally reported to be understandable, and there were few questions from parents or legal guardians seeking clarification of individual questions. If ordinal scale questions were answered between two values, the respondents were contacted again. If they continued to choose an intermediate value, the more severe phenotype was chosen. Regression was defined in the questionnaire as the loss of previously learned skills. None of the participants (PMS and healthy individuals) had an infection at the time of blood collection. Healthy individuals were only enrolled in the study if they had no neurological or psychological problems in their lives.

According to the genetic report subjects with PMS were grouped by genotype into terminal deletions (D = deletions with SHANK3 haploinsufficiency), interstitial deletions without SHANK3 haploinsufficiency (I), or *SHANK3*-associated mutations (V = variants), including pathogenic *SHANK3* variants, *SHANK3* intragenic deletions, or breakpoint mutations in the *SHANK3* gene with translocation.

### Statistical analysis

#### Analyses of mouse and human data for SHANK3 and beta-synuclein expression

Data are displayed as mean ± standard error of the mean (SEM). Statistical analysis and graphs were generated using GraphPad Prism (version 9.4.1, GraphPad, San Diego, California, US).

After checking for outliers, normal distribution was tested using the Shapiro-Wilk test. Based on the number of comparisons and the pattern of the data distribution, an appropriate statistical test was used to analyze the data.

Statistical tests were two-tailed with a significance levels (p-values) of a ≤ 0.05 with 95% confidence interval. The significance level was displayed as follow: *p ≤ 0.05, **p ≤ 0.01, ***p ≤ 0.001, ****p ≤ 0.0001.

For all graphic data, n indicates number of biological replicates. The n values are reported in the figure legends.

#### Analysis of subgroup differences

To assess whether the group differences in potential biomarker values (SHANK3 and beta-synuclein) were independent of potential confounders, the linear regression analyses using ordinary least squares (OLS) were performed. Levels of two biological measurements were modeled in relation to group status (PMS vs. Control), age, and sex. Influential covariates were further tested via within-group Pearson’s correlation analyses. Where applicable, these variables were included as adjustment factors in other models.

Since the SHANK3 protein expression was quantified via the analysis of Western blot bands and normalized to total protein per lane, SHANK3 values had no absolute unit and varied across an arbitrary scale. Therefore, SHANK3 was standardized (z-scored) prior to all regression analyses to enable interpretable effect estimates, opposed to beta-synuclein, which raw concentrations (pg/ml) were used to preserve unit-based explainability.

Furthermore, to account for genetic heterogeneity within the PMS cohort, additional multiple linear regression (MLR) models were fitted including genotype as a covariate (Deletion, Ring, Variants). The performance of each model was summarized using adjusted R², and significance of individual predictors was evaluated using Wald statistics (t-values) at a threshold of p < 0.05. Additionally, the genotype-specific variation in original-scale SHANK3 levels was first tested with a Kruskal–Wallis analysis (α = 0.05), in GraphPad Prism. Pairwise contrasts of z-scored SHANK3 and beta-synuclein across PMS genotypes were then evaluated with Tukey’s honest significant difference procedure, adjusted for multiple comparisons, and reported relative to the control group. Analyses were conducted in Python within a fully reproducible workflow unless otherwise noted.

#### Outlier identification

Outliers were identified using six methods: z-score thresholding ( | z | > 3), interquartile range (IQR., 1.5 × IQR), median absolute deviation (MAD., 3 × MAD), Grubbs’ test (α = 0.05), Dixon’s Q test (two-sided, n ≤ 30), and isolation forest (contamination = 0.1). These outlier detectors were selected to capture both parametric and non-parametric irregularities in case of small sample size. A majority-voting approach was used, in which data points flagged by at least five methods were excluded as outliers from the corresponding dataset, minimizing bias from implementing just a single detection strategy.

In the SHANK3 PMS dataset, one outlier was identified (code P5, see Supplementary Table), and another excluded due to poor western blot quality. Exclusions were limited to the respective dataset.

In the beta-synuclein control group, one sample (code C24) was consistently flagged as an outlier by all six detection models. The exclusion was limited to this dataset.

Code P7 was not included in any of the analysis due to the genetic status as PMS *SHANK3*-unrelated.

#### Analyses of the human phenotypic data in relation biomarker levels

Internal consistency of the questionnaire was assessed separately for binary and ordinal variables. For the binary subset, it was calculated using the Kuder–Richardson Formula 20. For the ranked questions, the Cronbach’s alpha was used.

The multiple-choice question on behavior problems was rearranged into separate binary-coded variables. All Likert scale variables were uniformly recoded to a 0–2 range to align the scoring across variables.

Nine questionnaire items that had at least five non-zero responses were kept for phenotypic analysis (five questions were removed).

Associations between the measurements of interest and behavioral/clinical phenotypes obtained from the questionnaire were investigated using bootstraped regression (1000 iterations). The models were selected based on measurement type: ordinal logistic regression for Likert scale features, and regularized binary logistic regression for dichotomous variables. Each model was run both with and without adjustment for PMS genotypes groups. To further explore within-genotype patterns, point-biserial correlations (for SHANK3) and Spearman rank correlations (for beta-synuclein) were computed separately within each genotype subgroup per significant phenotype.

To provide group-level effect sizes, additional comparisons were performed for the variables that showed significant associations with z-scored SHANK3 expression or plasma beta-synuclein levels in regression models. For these analyses, ordinal predictors were converted to binary, with adjacent levels combined based on clinical interpretability. Welch’s t-tests were used for group comparisons, and effect sizes were reported as Cohen’s d.

Lastly, to assess the potential relationship between two markers, unstandardized SHANK3 expression and plasma beta-synuclein concentrations were correlated using Spearman’s rank correlation, and z-scored SHANK3 and z-scored beta-synuclein were combined into regression model as interactive terms to control for shared influence.

## Results

### Peripheral blood mononuclear cells isolated from mice express SHANK3

**Fig. 1:**
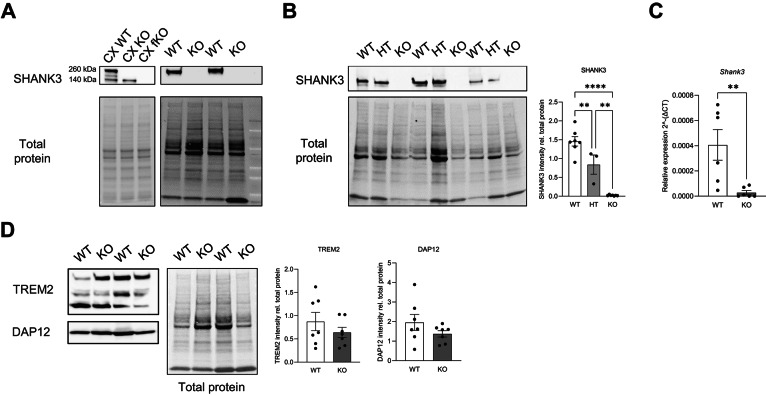
Shank3 expression in mice peripheral blood mononuclear cells. **A** Example of Shank3 expression in WT, Shank3Δ11-/- (KO) and Shank3 fullKO (fKO) cortical synaptosomal fraction (5 µg) and in peripheral blood mononuclear cells (40 µg) from WT and Shank3Δ11-/- (KO) adult mice. **B** Representative western blot images and relative SHANK3 quantification from peripheral blood mononuclear cells isolated from Shank3 WT, HT and KO adult mice. Protein expression was normalized to the respective total protein levels. Data were analyzed by one-way ANOVA; n = 7 WT, n = 3 Shank3∆11 + /−, n = 7 Shank3∆11-/-. ** p ≤ 0.01; **** p ≤ 0.0001. **C** Shank3 mRNA expression from PBMCs of WT and Shank3∆11-/- adult mice. Data were analyzed by two-tailed Mann-Whitney test; n = 6 WT, n = 6 Shank3∆11-/-. ** p ≤ 0.01. **D** Representative western blot images and relative protein quantification from peripheral blood mononuclear cells isolated from adult mice. Protein expression was normalized to the respective total protein levels. Data obtained from the quantification of DAP12 were analyzed by unpaired, two-tailed Student’s t-test with Welch’s correction; TREM2 was analyzed by unpaired, two-tailed Student’s t-test; n = 7 WT, n = 7 Shank3∆11-/-. **** p ≤ 0.0001.

To test whether SHANK3 can be measured in peripheral blood, we isolated peripheral blood mononuclear cells (PBMCs) from wild-type (WT) mice and Shank3Δ11 − /− mice. SHANK3 protein was first assessed by western blot, using mouse cortex lysates as a positive control (Fig. 1A). In WT PBMCs, we detected a SHANK3 band corresponding to the high-molecular-weight isoform, whereas no SHANK3 signal was observed in Shank3Δ11 − /− PBMCs, supporting assay specificity (Fig. 1A). Quantification confirmed a significant reduction of SHANK3 protein in Shank3Δ11 − /− PBMCs, and intermediate levels were observed in Shank3Δ11 + /− mice, consistent with gene dosage (Fig. 1B).

To exclude that differences reflected broad shifts in myeloid composition or general PBMC alterations, we quantified TREM2 and its adaptor DAP12 as myeloid-associated proteins and found no significant differences across genotypes (Fig. 1D). In addition, to provide orthogonal validation at the transcript level and further exclude non-specific immunoreactivity, we quantified Shank3 mRNA in PBMCs by qPCR. Shank3 transcripts were detectable in WT PBMCs and significantly reduced in Shank3Δ11 − /− PBMCs (Fig. 1C). Together, these results demonstrate that SHANK3 is measurable in PBMCs and is reduced in a Shank3 loss-of-function context.

### SHANK3 levels are markedly reduced in PBMCs from individuals with PMS

**Fig. 2:**
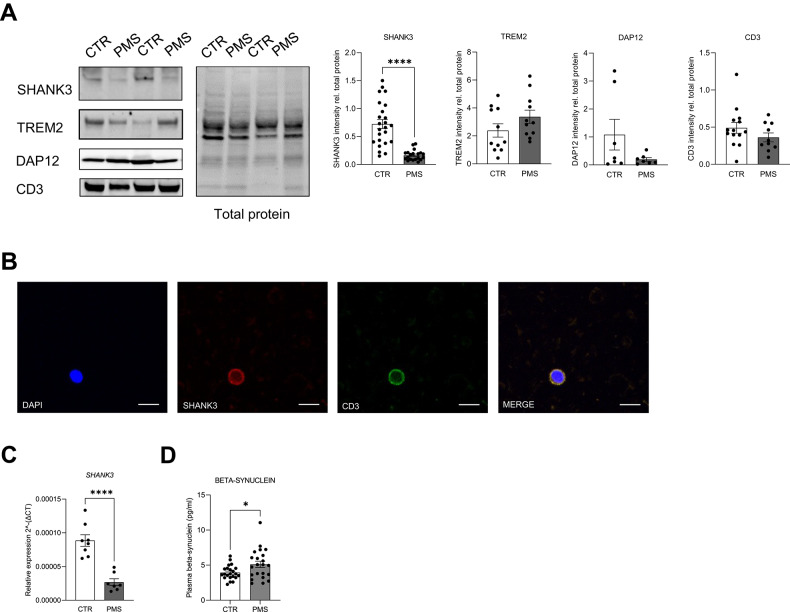
SHANK3 expression in human peripheral blood mononuclear cells. **A** Representative western blot images and relative protein quantification from peripheral blood mononuclear cells isolated from healthy donor and PMS patients. Protein expression was normalized to the respective total protein levels. Data obtained from the quantification of SHANK3 were analyzed by unpaired, two-tailed Student’s t-test with Welch’s correction; n = 24 controls, n = 20 patients; TREM2 was analyzed by unpaired, two-tailed Student’s t-test; n = 12 controls, n = 11 patients; DAP12 data were analyzed by two-tailed Mann-Whitney test; n = 7 controls, n = 7 patients; CD3 was analyzed by unpaired, two-tailed Student’s t-test; n = 14 controls, n = 10 patients. **** p ≤ 0.0001. **B** Immunocytochemistry of SHANK3 (red), CD3 (green) and DAPI (blue) in a peripheral blood smear of healthy donor sample. Scale bar: 10 µm. **C** *SHANK3* mRNA expression from PBMC of controls and PMS patients. Data were analyzed by unpaired, two-tailed Student’s t-test; n = 8 controls, n = 7 patients. **** p ≤ 0.0001. **D** Plasma concentration of beta-synuclein in healthy donors and PMS patient samples. Data were analyzed by unpaired, two-tailed Student’s t-test with Welch’s correction; n = 22 controls, n = 22 patients; * p ≤ 0.05.

After establishing peripheral detectability in mice, we assessed SHANK3 expression in PBMCs from individuals with PMS and healthy controls (Supplementary Table 1). SHANK3 was evaluated using complementary approaches (western blot, immunostaining, and RT-qPCR). Western blot quantification revealed a robust reduction of SHANK3 protein in PMS PBMCs, with a mean decrease of ~77% compared to controls (Fig. 2A). As expected for heterozygous SHANK3 alterations, SHANK3 remained detectable in PMS samples but was markedly reduced relative to controls.

To test whether the group difference could be explained by demographic covariates, we performed an OLS regression model controlling for sex and age. Z-scored SHANK3 levels remained significantly lower in PMS (coefficient = −1.35, p < 0.001), while sex and age were not significant predictors (sex p = 0.755; age p = 0.733). The model explained ~44% of the variance in SHANK3 levels (adjusted R² = 0.44).

Given genetic heterogeneity in PMS, we next included PMS genotype as a covariate (deletion, ring, variant). SHANK3 levels remained significantly reduced in PMS (coefficient = −1.05, p < 0.0001). Consistent with SHANK3 haploinsufficiency across PMS etiologies affecting SHANK3, all genotype groups showed significant reductions versus controls, while differences among PMS genotypes were not significant (Supplementary Fig. 1).

Moreover, Tukey’s HSD on z-scored SHANK3 levels revealed significant reductions versus controls for the Deletion ( − 1.34 SD, adjusted p = 0.0001), Ring ( − 1.54 SD, adjusted p = 0.0091), and Variants ( − 1.33 SD, adjusted p = 0.001) genotypes. No pairwise differences among the PMS genotypes reached significance (all adjusted p > 0.98).

To further support specificity and rule out trivial explanations related to PBMC yield/composition, we quantified TREM2/DAP12 and found no significant differences between PMS and controls (Fig. 2A). We additionally quantified CD3 as a T-cell marker to assess whether group differences could reflect gross differences in lymphocyte abundance; CD3 levels did not differ between groups (Fig. 2A). Immunostaining confirmed SHANK3 signal in human blood-derived cells (Fig. 2B), and RT-qPCR corroborated significantly reduced SHANK3 mRNA levels in PMS PBMCs (Fig. 2C).

Finally, to address rare PMS cases without SHANK3 involvement, we analyzed an internal comparison sample from an individual with a 22q13 deletion not encompassing SHANK3. In this case, SHANK3 protein and mRNA levels were comparable to controls (Supplementary Fig. 1), consistent with the expectation that SHANK3 reduction is linked to SHANK3-inclusive PMS genotypes. Collectively, these findings demonstrate that SHANK3 is measurable in human PBMCs and is specifically reduced in PMS cases affecting SHANK3.

### Plasma beta-synuclein is elevated in PMS and normalizes with mGlu5 modulation in Shank3 mice

To identify an additional liquid biomarker with potential relevance to synaptic dysfunction [21, 23–25, 28] and treatment response, we measured plasma beta-synuclein using immunoprecipitation mass spectrometry. Beta-synuclein levels were significantly increased in PMS compared to controls (Fig. 2D).

To evaluate potential confounding, we fit an OLS model adjusting for sex and age. Beta-synuclein remained significantly higher in PMS (coefficient = +0.98, p = 0.044). Age was a significant negative predictor (p = 0.004), indicating lower beta-synuclein with increasing age, while sex was not significant (p = 0.34). This model explained ~24% of the variance (adjusted R² = 0.24). Consistently, within the PMS group beta-synuclein showed a moderate negative correlation with age (Pearson r = −0.54, p = 0.009), whereas no significant age relationship was observed in controls (Pearson r = +0.26, p = 0.337). In a follow-up model including age and PMS genotype, the PMS-control group difference was attenuated and did not reach conventional significance (coefficient = +0.72, p = 0.06); post-hoc genotype comparisons were not significant, although all PMS genotype groups showed elevated mean levels versus controls.

**Fig. 3:**
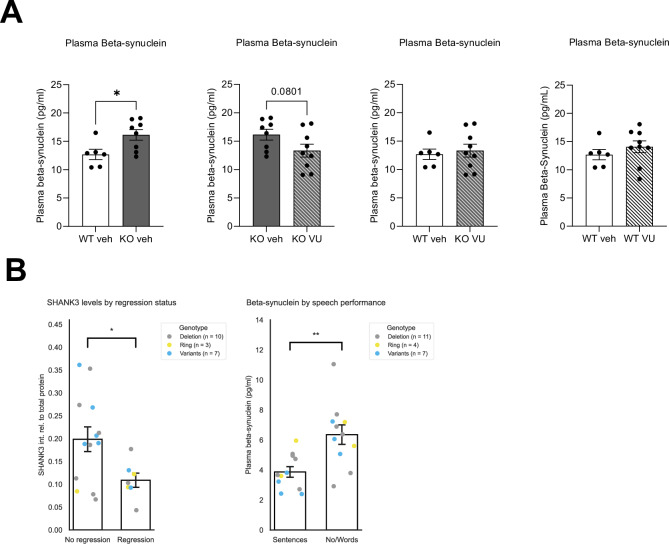
Dynamic changes of SHANK3 and beta-synuclein biomarkers. **A** Level of beta-synuclein isolated from plasma of WT and *Shank3*Δ11-/- adult mice after pharmacological treatment. Data were analyzed by unpaired, two-tailed Student’s t-test; n = 6 WT vehicle, n = 8 *Shank3*∆11-/- vehicle, n = 9 *Shank3*∆11-/- VU. * p ≤ 0.05. **B** Display of results of SHANK3 (unstandardized) and the occurrence of regression (left; Welch’s t = 2.8, p = 0.01, |Cohen’s d | = 1.20) and beta-synuclein levels in PMS individuals with different degrees of speech impairment (right; Welch’s t = 3.36, p = 0.0041, |Cohen’s d | = 1.43). Data were analyzed by a Welch’s t-test. * p ≤ 0.05, ** p ≤ 0.01.

To test translational relevance and explore whether beta-synuclein might function as a pharmacodynamic marker, we evaluated plasma beta-synuclein in Shank3Δ11 − /− mice. Shank3Δ11 − /− mice exhibited elevated plasma beta-synuclein relative to WT (Fig. 3A, left). We then treated mice with VU0409551, a positive allosteric modulator of mGlu5 previously shown to ameliorate molecular/behavioral deficits in Shank3 models [26]. Following 10 days of chronic treatment, plasma beta-synuclein was significantly reduced in Shank3Δ11 − /− mice and shifted toward WT levels (Fig. 3A). The same treatment showed no effect in WT mice (Fig. 3A) These findings support beta-synuclein as a candidate treatment-responsive plasma biomarker in a Shank3 context.

### Lower PBMC SHANK3 levels are associated with developmental regression

We tested associations between SHANK3 (z-scored) and behavioral/clinical phenotypes derived from the caregiver survey using bootstrap-based regression. Among the tested phenotypes, only regression (binary) was significantly associated with SHANK3 levels. Individuals with regression had significantly lower SHANK3 expression compared to those without regression (unadjusted for genotype: coefficient = −1.9, 95% CI [ − 4.04, −0.58], p = 0.002; bootstrapped n = 1000). This relationship persisted after controlling for PMS genotype (coefficient = −2.19, 95% CI [ − 6.83, −0.23], p = 0.03; bootstrapped n = 1000). Subgroup analyses by genotype did not yield statistically significant associations, although the variants subgroup showed a strong negative point-biserial correlation (r = −0.722) that did not reach significance (p = 0.06), consistent with limited power (n = 7). Direct comparison of unstandardized SHANK3 levels confirmed significantly lower SHANK3 in individuals with regression (Fig. 3B, left).

### Plasma beta-synuclein levels are associated with speech impairment severity

We next evaluated the relationship between beta-synuclein and clinical phenotypes using bootstrap-based ordinal regression controlling for age. Only speech was significantly associated with beta-synuclein levels. Higher ordinal speech scores were linked to lower beta-synuclein (unadjusted for genotype: coefficient = −0.81, CI [ − 1.56, −0.28], p = 0.0001; bootstrapped n = 997), and the association remained after adjusting for genotype (coefficient = −0.83, CI [ − 1.64, −0.25], p = 0.002; bootstrapped n = 997). Genotype-stratified analyses showed one significant association within the variants subgroup (Spearman r = −0.84, p = 0.019).

To facilitate interpretability and estimate effect size, we grouped participants by functional speech: individuals using sentences (group 1) versus those with no speech or only single words (group 2). Beta-synuclein was significantly higher in group 2 with a very large effect size (Fig. 3B, right).

### Combined models of SHANK3 and beta-synuclein

SHANK3 and beta-synuclein showed a weak, non-significant negative correlation (Spearman r = −0.146, p = 0.5395). We nevertheless fit combined models to explore complementary predictive information. In show severity, beta-synuclein showed a significant negative association (p = 0.041), whereas SHANK3 and the interaction term were not significant. For regression, SHANK3 showed a negative effect (p = 0.05) and the interaction term showed a suggestive trend (p = 0.082). These exploratory results are consistent with partially distinct biological information captured by the two measures and motivate validation in larger longitudinal cohorts.

## Discussion

Phelan–McDermid syndrome (PMS) is a rare, genetically defined neurodevelopmental disorder most commonly caused by deletions or pathogenic variants affecting SHANK3 at 22q13. In routine care, PMS is ultimately confirmed by genetic testing [10, 29, 30] once clinically suspected [28]. Therefore, the central translational challenge in PMS is not the development of alternative diagnostic screening tests, but rather the lack of objective, scalable biomarkers that can support patient stratification, inform prognosis, and provide monitoring and pharmacodynamic endpoints for therapeutic trials. This need is increasingly urgent given the growing number of interventional programs in PMS [31] and the limitations of behavioral outcome measures, which are often variable, age-dependent, and sensitive to contextual factors.

In this pilot study, we identify two complementary blood-based biomarker candidates: SHANK3 protein and mRNA in PBMCs, and plasma beta-synuclein. A major advantage of blood-based measures is the feasibility of repeated sampling with minimal burden, enabling longitudinal designs that are essential for understanding individual developmental trajectories and treatment effects [32–37].

### PBMC SHANK3 as a target-engagement and monitoring biomarker

We demonstrate that SHANK3 is detectable in PBMCs and is robustly reduced in both a Shank3 loss-of-function mouse model and individuals with PMS. In humans, PBMC SHANK3 protein levels were reduced on average by ~77% compared with controls, consistent with SHANK3 haploinsufficiency in most PMS genotypes. Importantly, this reduction remained significant after adjusting for age and sex, and SHANK3 levels were comparably reduced across PMS genotypes that involve SHANK3. Conversely, an internal comparison sample with a 22q13 deletion not involving SHANK3 showed SHANK3 levels comparable to controls, supporting biological and analytic specificity.

Because PBMCs represent a heterogeneous mixture of immune cell types, we assessed whether the observed SHANK3 reduction could reflect broad differences in immune composition or sample quality rather than SHANK3 biology. Levels of TREM2/DAP12 did not differ between groups, and CD3—used as a coarse marker for T-lymphocyte abundance—also showed no group differences, arguing against major shifts in PBMC yield or gross lymphocyte composition as the primary explanation [38]. Immunostaining further supported detectable SHANK3 signal in blood-derived cells, although future work using cell-type-specific markers and sorted immune subsets will be needed to identify the precise cellular sources and to determine whether PMS involves subtle immune compositional changes that could influence PBMC-based measures. However, based on previous reports of SHANK expression in immune cells [39] and clinical indications that PMS may be associated with increased susceptibility to infection [40–45], it is conceivable that altered SHANK3 expression impacts immune function.

A key translational implication is that PBMC SHANK3 is best viewed as a target-engagement/monitoring biomarker rather than a diagnostic screening tool. This framing is particularly relevant given the availability of genomic confirmation for PMS. Consistent with this intended use-case, we observed that lower PBMC SHANK3 levels were associated with the presence of developmental regression, suggesting potential value for prognosis or stratification. At the same time, we emphasize that peripheral SHANK3 levels may not directly mirror CNS SHANK3 levels. Establishing peripheral–central correspondence (including age-stratified designs and paired peripheral/CNS measurements in model systems) is an important next step.

### Plasma beta-synuclein as a candidate phenotypic and pharmacodynamic marker

Given the synaptic biology of PMS and the need for trial-ready markers, we also evaluated plasma beta-synuclein, a neuron-enriched synaptic protein measurable in blood using sensitive targeted proteomic approaches and previously implicated as a marker of synaptic pathology in other neurological contexts [22–25, 46–48]. We found a modest but significant elevation of plasma beta-synuclein in PMS compared with controls. In regression models, age emerged as a relevant covariate, and within the PMS group beta-synuclein showed an inverse relationship with age, underscoring the need for age-stratified and longitudinal validation—particularly in pediatric cohorts. Importantly, beta-synuclein levels were associated with speech impairment severity, suggesting potential utility for phenotypic stratification and clinical trial enrichment strategies.

To explore whether beta-synuclein might also function as a treatment-responsive biomarker, we back-translated this measure in the Shank3 mouse model. Beta-synuclein was elevated in Shank3Δ11 − /− plasma and was normalized by chronic treatment with the mGlu5 positive allosteric modulator VU0409551. We chose mGlu5 modulation because synaptic glutamatergic signaling is mechanistically connected to SHANK3-related synaptic scaffolding and because mGlu5-directed interventions have shown beneficial effects in Shank3-related models [26]. While these findings do not establish clinical efficacy, they support beta-synuclein as a plausible pharmacodynamic readout of pathway engagement and treatment-related normalization in a Shank3 context. This is particularly relevant given the phenotypic variability and individual developmental trajectories characteristic of PMS [19]. Regression—the loss of previously acquired skills—is commonly reported among individuals with PMS [49, 50], and several pharmaceutical companies are actively pursuing causal therapies, including gene therapy, SHANK3 upregulation, and synaptic modulation approaches (see clinicaltrials.gov) [51, 52].

### Translation, limitations, and future directions

Several limitations should be considered. First, this is a pilot cohort; larger multi-center cohorts are required to confirm effect sizes, refine covariate adjustment (including medication, comorbidities, time-of-day and other pre-analytic variables), and determine reproducibility across sites and platforms. Second, our human cohort is adult-enriched; given the age dependence observed for beta-synuclein and the developmental nature of PMS, validation in pediatric and longitudinal cohorts is essential. Third, PBMC-based SHANK3 quantification currently relies on research-grade methods; while our data support specificity (including KO controls and orthogonal mRNA validation), translation to higher-throughput formats will require further assay development and standardization. Finally, clinical phenotyping was derived from caregiver-reported survey data, which can introduce noise and limit resolution; future work should integrate standardized clinical instruments and objective measures where feasible.

Despite these limitations, our findings provide initial evidence for two accessible blood-based measures that capture complementary aspects of PMS biology: PBMC SHANK3 as a feasible marker related to SHANK3 haploinsufficiency and regression status, and plasma beta-synuclein as a neuron-enriched marker associated with speech impairment and responsive to pathway modulation in a Shank3 model. Together, these biomarkers may help enable more objective monitoring of disease course and therapeutic response in PMS, supporting the design and interpretation of ongoing and future interventional studies.

### Weblinks

**ClinicalTrials.gov**: https://clinicaltrials.gov/. **Phelan-McDermid Guideline**: https://ern-ithaca.eu/guidelines/ern-ithaca-guidelines/phelan-mcdermid-guideline/.

## Supplementary information

Figure Suppl 1

Suppl Table 1

Legend

## Data Availability

The data that support the findings of this study are available from the corresponding authors, upon reasonable request.

## References

[1] Phelan K, McDermid HE. The 22q13.3 deletion syndrome (Phelan-McDermid syndrome). Mol Syndromol. 2012;2:186–201. 22670140 10.1159/000334260 PMC3366702

[2] Phelan MC, Rogers RC, Saul RA, Stapleton GA, Sweet K, McDermid H, et al. 22q13 deletion syndrome. Am J Med Genet. 2001;101:91–9. 11391650 10.1002/1096-8628(20010615)101:2<91::aid-ajmg1340>3.0.co;2-c

[3] Denayer A, Van Esch H, de Ravel T, Frijns JP, Van Buggenhout G, Vogels A, et al. Neuropsychopathology in 7 patients with the 22q13 deletion syndrome: presence of bipolar disorder and progressive loss of skills. Mol Syndromol. 2012;3:14–20. 22855650 10.1159/000339119 PMC3398818

[4] Vitrac A, Leblond CS, Rolland T, Cliquet F, Mathieu A, Maruani A, et al. Dissecting the 22q13 region to explore the genetic and phenotypic diversity of patients with Phelan-McDermid syndrome. Eur J Med Genet. 2023;66:104732. 36822569 10.1016/j.ejmg.2023.104732

[5] Koza SA, Tabet AC, Bonaglia MC, Andres S, Anderlid BM, Aten E, et al. Consensus recommendations on counselling in Phelan-McDermid syndrome, with special attention to recurrence risk and to ring chromosome 22. Eur J Med Genet. 2023;66:104773. 37120077 10.1016/j.ejmg.2023.104773

[6] Disciglio V, Lo Rizzo C, Mencarelli MA, Mucciolo M, Marozza A, Di Marco C, et al. Interstitial 22q13 deletions not involving SHANK3 gene: a new contiguous gene syndrome. Am J Med Genet A. 2014;164A:1666–76. 24700646 10.1002/ajmg.a.36513

[7] Simenson K, Õiglane-Shlik E, Teek R, Kuuse K, Õunap K. A patient with the classic features of Phelan-McDermid syndrome and a high immunoglobulin E level caused by a cryptic interstitial 0.72-Mb deletion in the 22q13.2 region. Am J Med Genet A. 2014;164:806–9. 10.1002/ajmg.a.36358 24375995

[8] Li S, Xi KW, Liu T, Zhang Y, Zhang M, Zeng LD, et al. Fraternal twins with Phelan-McDermid syndrome not involving the SHANK3 gene: case report and literature review. BMC Med Genomics. 2020;13:146. 33023580 10.1186/s12920-020-00802-0 PMC7539423

[9] Wilson HL, Crolla JA, Walker D, Artifoni L, Dallapiccola B, Takano T, et al. Interstitial 22q13 deletions: genes other than SHANK3 have major effects on cognitive and language development. Eur J Hum Genet. 2008;16:1301–10. 18523453 10.1038/ejhg.2008.107

[10] Phelan K, Boccuto L, Powell CM, Boeckers TM, van Ravenswaaij-Arts C, Rogers RC, et al. Phelan-McDermid syndrome: a classification system after 30 years of experience. Orphanet J Rare Dis. 2022;17:27. 35093143 10.1186/s13023-022-02180-5 PMC8800328

[11] Roussignol G, Ango F, Romorini S, Tu JC, Sala C, Worley PF, et al. Shank expression is sufficient to induce functional dendritic spine synapses in aspiny neurons. J Neurosci. 2005;25:3560–70. 15814786 10.1523/JNEUROSCI.4354-04.2005 PMC6725374

[12] Sala C, Vicidomini C, Bigi I, Mossa A, Verpelli C. Shank synaptic scaffold proteins: keys to understanding the pathogenesis of autism and other synaptic disorders. J Neurochem. 2015;135:849–58. 26338675 10.1111/jnc.13232

[13] Sheng M, Kim E. The Shank family of scaffold proteins. J Cell Sci. 2000;113:1851–56. 10806096 10.1242/jcs.113.11.1851

[14] Malara M, Lutz AK, Incearap B, Bauer HF, Cursano S, Volbracht K, et al. SHANK3 deficiency leads to myelin defects in the central and peripheral nervous system. Cell Mol Life Sci. 2022;79:371. 35726031 10.1007/s00018-022-04400-4 PMC9209365

[15] Jesse S, Müller HP, Schoen M, Asoglu H, Bockmann J, Huppertz HJ, et al. Severe white matter damage in SHANK3 deficiency: a human and translational study. Ann Clin Transl Neurol. 2020;7:46–58. 31788990 10.1002/acn3.50959 PMC6952316

[16] Durand CM, Betancur C, Boeckers TM, Bockmann J, Chaste P, Fauchereau F, et al. Mutations in the gene encoding the synaptic scaffolding protein SHANK3 are associated with autism spectrum disorders. Nat Genet. 2007;39:25–7. 17173049 10.1038/ng1933 PMC2082049

[17] Durand CM, Perroy J, Loll F, Perrais D, Fagni L, Bourgeron T, et al. SHANK3 mutations identified in autism lead to modification of dendritic spine morphology via an actin-dependent mechanism. Mol Psychiatry. 2012;17:71–84. 21606927 10.1038/mp.2011.57 PMC3252613

[18] Leblond CS, Nava C, Polge A, Gauthier J, Huguet G, Lumbroso S, et al. Meta-analysis of SHANK mutations in autism spectrum disorders: a gradient of severity in cognitive impairments. PLoS Genet. 2014;10:e1004580. 25188300 10.1371/journal.pgen.1004580 PMC4154644

[19] Schön M, Lapunzina P, Nevado J, Mattina T, Gunnarsson C, Hadzsiev K, et al. Definition and clinical variability of SHANK3-related Phelan-McDermid syndrome. Eur J Med Genet. 2023;66:104754. 37003575 10.1016/j.ejmg.2023.104754

[20] Bauer HF, Delling JP, Bockmann J, Boeckers TM, Schön M. Development of sex- and genotype-specific behavioral phenotypes in a Shank3 mouse model for neurodevelopmental disorders. Front Behav Neurosci. 2022;16:1051175. 36699652 10.3389/fnbeh.2022.1051175 PMC9868822

[21] Oeckl P, Mayer B, Bateman RJ, Day GS, Fox NC, Huey ED, et al. Early increase of the synaptic blood marker β-synuclein in asymptomatic autosomal dominant Alzheimer’s disease. Alzheimers Dement. 2025;21:e70146. 40207431 10.1002/alz.70146 PMC11982912

[22] Oeckl P, Halbgebauer S, Anderl-Straub S, von Arnim CAF, Diehl-Schmid J, Froelich L, et al. Targeted mass spectrometry suggests beta-synuclein as synaptic blood marker in Alzheimer’s disease. J Proteome Res. 2020;19:1310–8. 32101007 10.1021/acs.jproteome.9b00824

[23] Oeckl P, Janelidze S, Halbgebauer S, Stomrud E, Palmqvist S, Otto M, et al. Higher plasma β-synuclein indicates early synaptic degeneration in Alzheimer’s disease. Alzheimers Dement. 2023;19:5095–102. 37186338 10.1002/alz.13103

[24] Oeckl P, Anderl-Straub S, Danek A, Diehl-Schmid J, Fassbender K, Fliessbach K, et al. Relationship of serum beta-synuclein with blood biomarkers and brain atrophy. Alzheimers Dement. 2023;19:1358–71. 36129098 10.1002/alz.12790

[25] Barba L, Abu Rumeileh S, Bellomo G, Paolini Paoletti F, Halbgebauer S, Oeckl P, et al. Cerebrospinal fluid β-synuclein as a synaptic biomarker for preclinical Alzheimer’s disease. J Neurol Neurosurg Psychiatry. 2023;94:83–6. 35944974 10.1136/jnnp-2022-329124

[26] Giona F, Beretta S, Zippo A, Stefanoni A, Tomasoni Z, Vicidomini C, et al. Shank3 modulates Rpl3 expression and protein synthesis via mGlu5: implications for Phelan McDermid syndrome. Mol Psychiatry. 2025;30:3599–614. 10.1038/s41380-025-02947-9. 40089604 10.1038/s41380-025-02947-9 PMC12240844

[27] Vicidomini C, Ponzoni L, Lim D, Schmeisser MJ, Reim D, Morello N, et al. Pharmacological enhancement of mGlu5 receptors rescues behavioral deficits in SHANK3 knock-out mice. Mol Psychiatry. 2017;22:689–702. 27021819 10.1038/mp.2016.30 PMC5014121

[28] Cammarata-Scalisi F, Callea M, Martinelli D, Willoughby CE, Cárdenas Tadich A, Araya Castillo M, et al. Clinical and genetic aspects of Phelan-McDermid syndrome: an interdisciplinary approach to management. Genes. 2022;13:504. 35328058 10.3390/genes13030504 PMC8955098

[29] Peters DG, Yatsenko SA, Surti U, Rajkovic A. Recent advances of genomic testing in perinatal medicine. Semin Perinatol. 2015;39:44–54. 25444417 10.1053/j.semperi.2014.10.009 PMC4883661

[30] Stessman HA, Bernier R, Eichler EE. A genotype-first approach to defining the subtypes of a complex disease. Cell. 2014;156:872–77. 24581488 10.1016/j.cell.2014.02.002 PMC4076166

[31] Dawson G, Jones EJH, Merkle K, Venema K, Lowy R, Faja S, et al. Early behavioral intervention is associated with normalized brain activity in young children with autism. J Am Acad Child Adolesc Psychiatry. 2012;51:1150–59. 23101741 10.1016/j.jaac.2012.08.018 PMC3607427

[32] Loth E, Spooren W, Ham LM, Isaac MB, Auriche-Benichou C, Banaschewski T, et al. Identification and validation of biomarkers for autism spectrum disorders. Nat Rev Drug Discov. 2016;15:70–3. 26718285 10.1038/nrd.2015.7

[33] Sahin M, Sweeney JA, Jones SR. Editorial: biomarkers to enable therapeutics development in neurodevelopmental disorders. Front Integr Neurosci. 2020;14:616641. 33262695 10.3389/fnint.2020.616641 PMC7686575

[34] Sullivan PF, Fan C, Perou CM. Evaluating the comparability of gene expression in blood and brain. Am J Med Genet B Neuropsychiatr Genet. 2006;141B:261–68. 16526044 10.1002/ajmg.b.30272

[35] Rollins B, Martin MV, Morgan L, Vawter MP. Analysis of whole genome biomarker expression in blood and brain. Am J Med Genet B Neuropsychiatr Genet. 2010;153B:919–36. 20127885 10.1002/ajmg.b.31062 PMC3098564

[36] Nardo G, Pozzi S, Pignataro M, Lauranzano E, Spano G, Garbelli S, et al. Amyotrophic lateral sclerosis multiprotein biomarkers in peripheral blood mononuclear cells. PLoS One. 2011;6:e25545. 21998667 10.1371/journal.pone.0025545 PMC3187793

[37] Pansarasa O, Garofalo M, Scarian E, Dragoni F, Garau J, Di Gerlando R, et al. Biomarkers in human peripheral blood mononuclear cells: the state of the art in amyotrophic lateral sclerosis. Int J Mol Sci. 2022;23:2580. 35269723 10.3390/ijms23052580 PMC8910056

[38] Kleiveland CR Peripheral blood mononuclear cells. In: Verhoeckx K, Cotter P, López-Expósito I, Kleiveland C, Lea T, Mackie A et al. eds. The Impact of Food Bioactives on Health: In Vitro and Ex Vivo Models. Cham: Springer, 2015:pp. 161–67. 29787039

[39] Redecker P, Bockmann J, Böckers TM. Expression of postsynaptic density proteins of the ProSAP/Shank family in the thymus. Histochem Cell Biol. 2006;126:679–85. 16758162 10.1007/s00418-006-0199-9

[40] De Rubeis S, Siper PM, Durkin A, Weissman J, Muratet F, Halpern D, et al. Delineation of the genetic and clinical spectrum of Phelan-McDermid syndrome caused by SHANK3 point mutations. Mol Autism. 2018;9:31. 29719671 10.1186/s13229-018-0205-9 PMC5921983

[41] Dhar SU, del Gaudio D, German JR, Peters SU, Ou Z, Bader PI, et al. 22q13.3 deletion syndrome: clinical and molecular analysis using array CGH. Am J Med Genet A. 2010;152A:573–81. 20186804 10.1002/ajmg.a.33253 PMC3119894

[42] Samogy-Costa CI, Varella-Branco E, Monfardini F, Ferraz H, Fock RA, Barbosa RHA, et al. A Brazilian cohort of individuals with Phelan-McDermid syndrome: genotype-phenotype correlation and identification of an atypical case. J Neurodev Disord. 2019;11:13. 31319798 10.1186/s11689-019-9273-1 PMC6637483

[43] Xu N, Lv H, Yang T, Du X, Sun Y, Xiao B, et al. A 29 Mainland Chinese cohort of patients with Phelan-McDermid syndrome: genotype-phenotype correlations and the role of SHANK3 haploinsufficiency in the important phenotypes. Orphanet J Rare Dis. 2020;15:335. 33256793 10.1186/s13023-020-01592-5 PMC7708101

[44] Soorya L, Leon J, Trelles MP, Thurm A. Framework for assessing individuals with rare genetic disorders associated with profound intellectual and multiple disabilities (PIMD): the example of Phelan McDermid syndrome. Clin Neuropsychol. 2018;32:1226–55. 29265961 10.1080/13854046.2017.1413211 PMC6417924

[45] Breen MS, Fan X, Levy T, Pollak RM, Collins B, Osman A, et al. Large 22q13.3 deletions perturb peripheral transcriptomic and metabolomic profiles in Phelan-McDermid syndrome. HGG Adv. 2022;4:100145. 36276299 10.1016/j.xhgg.2022.100145 PMC9579712

[46] Oeckl P, Metzger F, Nagl M, von Arnim CAF, Halbgebauer S, Steinacker P, et al. Alpha-, beta-, and gamma-synuclein quantification in cerebrospinal fluid by multiple reaction monitoring reveals increased concentrations in Alzheimer’s and Creutzfeldt-Jakob disease but no alteration in synucleinopathies. Mol Cell Proteom. 2016;15:3126–38. 10.1074/mcp.M116.059915 PMC5054339 27507836

[47] Bergström S, Remnestål J, Yousef J, Olofsson J, Markaki I, Carvalho S, et al. Multi-cohort profiling reveals elevated CSF levels of brain-enriched proteins in Alzheimer’s disease. Ann Clin Transl Neurol. 2021;8:1456–70. 34129723 10.1002/acn3.51402 PMC8283172

[48] Halbgebauer S, Oeckl P, Steinacker P, Yilmazer-Hanke D, Anderl-Straub S, von Arnim C, et al. Beta-synuclein in cerebrospinal fluid as an early diagnostic marker of Alzheimer’s disease. J Neurol Neurosurg Psychiatry. 2021;92:349–56. 33380492 10.1136/jnnp-2020-324306

[49] Reierson G, Bernstein J, Froehlich-Santino W, Urban A, Purmann C, Berquist S, et al. Characterizing regression in Phelan McDermid syndrome (22q13 deletion syndrome). J Psychiatr Res. 2017;91:139–44. 28346892 10.1016/j.jpsychires.2017.03.010 PMC5469716

[50] Kohlenberg TM, Trelles MP, McLarney B, Betancur C, Thurm A, Kolevzon A. Psychiatric illness and regression in individuals with Phelan-McDermid syndrome. J Neurodev Disord. 2020;12:7. 32050889 10.1186/s11689-020-9309-6 PMC7014655

[51] van Ravenswaaij-Arts CMA, van Balkom IDC, Jesse S, Bonaglia MC. Editorial: Towards a European consensus guideline for Phelan-McDermid syndrome. Eur J Med Genet. 2023;66:104736. 36907549 10.1016/j.ejmg.2023.104736

[52] Srivastava S, Sahin M, Buxbaum JD, Berry-Kravis E, Soorya LV, Thurm A, et al. Updated consensus guidelines on the management of Phelan-McDermid syndrome. Am J Med Genet A. 2023;191:2015–44. 37392087 10.1002/ajmg.a.63312 PMC10524678

