# Supplementary figures and images for: SHANK3 and beta-synuclein are novel blood-based biomarkers for the Phelan-McDermid Syndrome: a pilot study
Source: Transl Psychiatry. 2026 Mar 24;16:201. doi: 10.1038/s41398-026-03932-8 (PMC13039877; doi:10.1038/s41398-026-03932-8)

Suppl Fig1

A

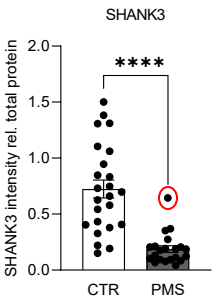

B

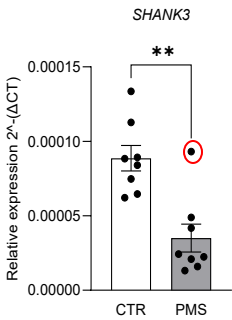

C

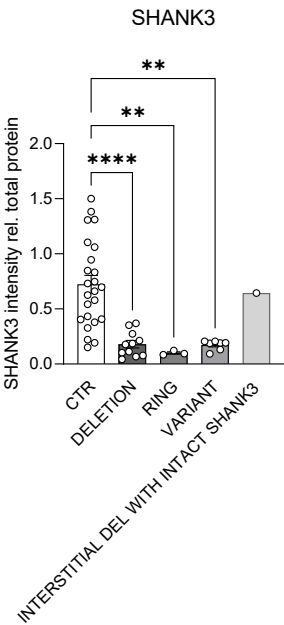

Supplement: Supplementary file 1 — Figure Suppl 1 [file 41398_2026_3932_MOESM1_ESM.pdf]
