# Supplementary material for: SHANK3 and beta-synuclein are novel blood-based biomarkers for the Phelan-McDermid Syndrome: a pilot study
Source: Transl Psychiatry. 2026 Mar 24;16:201. doi: 10.1038/s41398-026-03932-8 (PMC13039877; doi:10.1038/s41398-026-03932-8)
Supplement: Supplementary file 2 — Suppl Table 1 [file 41398_2026_3932_MOESM2_ESM.pdf]

Suppl. Table 1 – Participants of the study

SAMPLES	SEX	AGE	CONDITION
CTR1	F	33	healty
CTR2	F	33	healty
CTR3	M	30	healty
CTR4	M	26	healty
CTR5	M	30	healty
CTRB	F	19	healty
CTRC	F	20	healty
CTRE	F	24	healty
CTRF	F	26	healty
CTRG	F	20	healty
CTRI	F	19	healty
CTRL	F	20	healty
CTR M	M	61	healty
CTR O	M	24	healty
CTR P	M	26	healty
CTR Q	M	20	healty
CTR R	M	20	healty
CTR S	M	29	healty
CTR U	M	19	healty
CTR V	M	25	healty
CTR W	F	20	healty
CTR X	F	32	healty
CTR Y	F	22	healty
CTR Z	F	27	healty

SAMPLES	SEX	AGE	CONDITION
P1	M	15	PMS - V
P5	M	62	PMS - (breakpoint)
P15	M	15	PMS - R
P36	F	22	PMS -D
P16	F	29	PMS - D
P32	M	31	PMS -D
P41	M	17	PMS -V
P46	F	18	PMS - V
P49	F	15	PMS - I
P65	M	16	PMS - D
P57	F	43	PMS - D
P59	M	15	PMS -V
P48	F	18	PMS -D
P62	F	19	PMS -D
P67	F	37	PMS -R
P72	F	30	PMS -D
P69	M	16	PMS -V
P24	F	11	PMS -R
P39	F	6	PMS - D
P27	M	5	PMS -D
P17	M	15	PMS -V
P42	M	17	PMS -R
P64	F	15	PMS -D

LEGEND (genetic infos):	
V	Variant
D	Deletion
R	Ring (can be also be counted as D)
I	(Interstitial deletion with 2 intact SHANK3 alleles)
