# Supplementary material for: SHANK3 and beta-synuclein are novel blood-based biomarkers for the Phelan-McDermid Syndrome: a pilot study
Source: Transl Psychiatry. 2026 Mar 24;16:201. doi: 10.1038/s41398-026-03932-8 (PMC13039877; doi:10.1038/s41398-026-03932-8)
Supplement: Supplementary file 3 — Legend [file 41398_2026_3932_MOESM3_ESM.docx]

SUPPLEMENTARY MATERIAL

Supplementary Table 1. PMS individuals and healthy controls.

F = female, M = male. D = terminal deletions with SHANK3 haploinsufficiency, I = interstitial deletions without SHANK3 haploinsufficiency, V = *SHANK3*-associated mutations, including pathogenic *SHANK3* variants, *SHANK3* intragenic deletions, or breakpoint mutations in the *SHANK3* gene with translocation.

Supplementary Fig. 1. A Representative western blot images and relative protein quantification from peripheral blood mononuclear cells isolated from healthy donor and PMS patients. Protein expression was normalized to the respective total protein levels. Data obtained from the quantification of SHANK3 were analyzed by unpaired, two-tailed Student’s t-test with Welch’s correction; n= 24 controls, n= 21 patients. Value for the patient with intact Shank3 is highlighted with a red circle. **** p ≤ 0.0001. B *SHANK3* mRNA expression from PBMC of controls and PMS patients. Data were analyzed by two-tailed Mann-Whitney test; n=8 controls, n=8 patients. Value for the patient with intact *SHANK3* is highlighted with a red circle. ** p ≤ 0.01. C SHANK3 quantification from peripheral blood mononuclear cells isolated from healthy donor and PMS patients. Values from patients with different mutations are compared to control samples. Data were analyzed by Kruskal-Wallis test; n=24 controls, n=11 patients with deletion, n=7 patients with a variant mutation, n=3 patients with a ring mutation, n=1 patient with intact *SHANK3*. ** p ≤ 0.01; **** p ≤ 0.0001.
